# The possible connection between neutrophil-to-high-density lipoprotein ratio and cerebral perfusion in clinically established corticobasal syndrome: a pilot study

**DOI:** 10.3389/fneur.2024.1464524

**Published:** 2024-10-03

**Authors:** Patryk Chunowski, Bartosz Migda, Natalia Madetko-Alster, Anna Migda, Michał Kutyłowski, Leszek Królicki, Piotr Alster

**Affiliations:** ^1^Department of Neurology, Medical University of Warsaw, Warsaw, Poland; ^2^Diagnostic Ultrasound Lab, Department of Pediatric Radiology, Medical University of Warsaw, Warsaw, Poland; ^3^Department of Endocrinology, Diabetology and Internal Medicine, Medical University of Warsaw, Warsaw, Poland; ^4^Department of Radiology, Mazovian Brodno Hospital, Warsaw, Poland; ^5^Department of Nuclear Medicine, Medical University of Warsaw, Warsaw, Poland

**Keywords:** NLR, PLR, NHR, atypical parkinsonism, SPECT, neuroinflammation

## Abstract

**Introduction:**

Progressive supranuclear palsy (PSP) and corticobasal syndrome (CBS) are tauopathic atypical parkinsonisms. Given their overlap in terms of clinical manifestation, there is growing interest in the mechanisms leading to these entities.

**Materials and methods:**

In total, 71 patients were included in the study, 19 of whom were clinically diagnosed with CBS, 37 with PSP, and 15 with Parkinson's disease (PD). The mean ages of the participants were 72.8, 72.9, and 64.0 years, respectively, and the disease duration varied from 3 to 6 years. Each individual underwent blood collection. Morphological and biochemical evaluation of blood samples was performed to analyze the neutrophil-to-lymphocyte ratio (NLR), platelet-to-lymphocyte ratio (PLR), and neutrophil-to-high-density lipoprotein ratio (NHR). A single-photon emission computed tomography (SPECT) with technetium-99m hexamethylpropyleneamine oxime (^99^Tc-HMPAO) tracer was used to assess perfusion in two regions of interest (ROI): the thalamus and insula. Using Pearson correlation to assess the linear relationship between NHR and perfusion in the insula and thalamus for CBS, PSP, and PD patients, the authors intended to verify possible correlations between NLR, PLR, and NHR and perfusion in the indicated ROIs.

**Results:**

The study revealed a negative linear correlation between NHR and perfusion of both the left (Insula L; R = −0.59) and right (Insula R; R = −0.58) insula regions. Similar to the insula, a linear correlation between NHR and activity in both the left (Thalamus L) and right (Thalamus R) thalamus regions in CBS subjects with a relatively stronger correlation in the right thalamus (R = −0.64 vs. R = −0.58) was found. These observations were not confirmed in PSP and PD patients.

**Conclusion:**

Simultaneously using non-specific parameters for peripheral inflammation (NLR, PLR, and NHR) and perfusion, SPECT may be an interesting beginning point for further analysis of inflammatory disease mechanisms. To the best of our knowledge, this is the first study to address the potential correlation between the peripheral neuroinflammatory markers NLR, PLR, and NHR and perfusion disturbances in particular ROIs.

## Introduction

Progressive supranuclear palsy (PSP) and corticobasal syndrome (CBS) are complex, clinically diverse conditions that are often referred to as “atypical Parkinsonian” disorders (1). PSP and CBS are very rare conditions; the prevalence of PSP is ~3–7 per 100,000, and the CBS prevalence ranges from 5 to 7 per 100,000 (2). The neuropathological identification of PSP relies on detecting neurofibrillary tangles and threads in the subcortical nuclei, along with the presence of tufted astrocytes. In addition, observations may include the presence of coiled oligodendroglia and diffuse cytoplasmic immunoreactivity in neuronal tissue (3). PSP manifests through substantial postural instability, recurrent falls, axial rigidity, cognitive dysfunction, and vertical supranuclear gaze palsy (4, 5). Clinically, CBS is characterized by asymmetric parkinsonism, limb apraxia, and cortical sensory deficits, accompanied by progressive dystonia, myoclonus, and alien limb phenomenon (6–8). CBS is associated with morphologically asymmetric cortical atrophy, variable basal ganglia, and nigral degeneration (9). CBS can be a manifestation of various pathologies, including corticobasal degeneration (CBD), PSP, frontotemporal dementia (FTD) (10), posterior cortical atrophy (11), and Alzheimer's disease (AD) (12–14). CBD is a neurodegenerative disorder in which a neuropathological evaluation can find abnormal neurons and glial cells (notably astrocytic plaques), tau protein accumulations in both the gray and white matter of the neocortex and striatum, along with swollen neurons, and localized loss of neurons in the neocortex and substantia nigra (6). CBD can clinically manifest through various syndromes, among which can include, apart from CBS, the non-fluent/agrammatic variant of primary progressive aphasia or frontal behavioral-spatial syndrome (11). They often manifest in individuals in their 60s or older, with some cases presenting in individuals in their 50s or even younger. Both diseases are classified as four-repeat tauopathies. Tau is encoded by the microtubule-associated protein tau (MAPT) gene, producing six protein isoforms that are tightly regulated. The inclusion or exclusion of exon 10 results in the formation of 4-repeat (4R) tau and 3-repeat (3R) tau, respectively (15). Various tauopathies' pathogenesis results from disruption of the 3R:4R tau ratio (16). It is implicated in several 4R diseases (other than PSP and CBD), such as FTLD-MAPT, argyrophilic grain disease (AGD), and globular glial tauopathy (GGT). AGD is most often found among patients over 80 years old as a single neurodegenerative condition or as a manifestation of AD or PSP (17). Another 4R tauopathy is a GGT that is divided into three types. The first type is indicative of a sporadic multiple system tauopathy associated with presenile dementia. The second type is distinguished by being more indicative of motor neuron disease, and the third type is a peculiar mix of two previous entities (18). There are many hypotheses attempting to explain the exact pathomechanism of the disease. Among the hypotheses concerning pathogenesis, inflammatory (19–21), vascular (19, 22, 23), and environmental (19, 22) theories were considered. The vascular hypothesis is based on brain hypoperfusion, which leads to neurodegeneration (19). The inflammatory hypothesis is linked with microglial activation. Microglial cells are part of the innate immune system and serve as the primary macrophages within the central nervous system (CNS). Astrocytes are the most prevalent cell type and can be found throughout all areas of the CNS. Physiologically, microglial cells provide physical and metabolic support to neurons, assist in detoxification, guide cell migration, and facilitate the regulation of metabolic energy. In response to a disease or injury, astrocytes experience a persistent activation known as astrogliosis. Similar to microglia, reactive astrocytes can develop a pro-inflammatory phenotype (24).

The inflammation is characterized by the activation of microglia and subsequent astrocyte response, accompanied by heightened cytokine expression and immune system mediators in both the cerebrospinal fluid (CSF) and the brain (25). The inflammatory hypothesis is linked to reactive astrocytes and microglia accumulation around amyloid deposits (4). The infiltration of peripheral immune cells influences microglia to adapt to a pro-inflammatory state, thereby accelerating disease progression. Microglia have the capacity to curb the spread of tau through phagocytosis. It can also intensify neurodegeneration by facilitating the distribution of these proteins (26). It is uncertain whether tau is the cause or the effect of the neuroinflammatory response (27). There is an increasing interest in peripheral inflammatory markers in the context of neurodegeneration. This research aimed to evaluate the possible correlation between peripheral inflammation and atrophic changes in certain regions of interest (ROIs) in PSP, CBS, and PD.

## Methods and data collection

### Study group

The analyzed group involved 71 patients: 19 with CBS, 37 with PSP, and 15 with Parkinson's disease (PD). Eligible patients diagnosed with CBS were identified and confirmed according to Armstrong's criteria (8). PSP individuals fulfilled the MDS criteria for PSP (5), and a PD diagnosis was established based on Postuma's clinical criteria. (28). The mean age of the groups was 72.8, 72.9, and 64.0 years, respectively. In the first group, there were eighteen women and one man; in the second group, there were eighteen women and nineteen men; while the last cohort was represented by seven women and eight men aged-matched in relation to the research group.

### Data collection

Neutrophil counts serve as parameters of inflammation (29, 30), and high-density lipoprotein cholesterol (HDL-C) is a component of atherosclerosis (29, 31). The neutrophil-to-lymphocyte ratio (NLR) can be used to assess the background of inflammatory illness (32, 33). The measure is derived by dividing the neutrophil count by the lymphocyte count in peripheral blood. NLR reflects the balance between acute and chronic inflammation, as shown by neutrophils, and adaptive immunity, as represented by lymphocytes (34).

Similarly, the platelet-to-lymphocyte ratio (PLR), determined by the ratio of platelet-to-lymphocyte counts in the blood, serves as an indicator of changes in the balance between the platelet count, which is linked to acute inflammatory responses and clot formation tendencies, and the lymphocyte count, reflecting the state of adaptive immunity (35). The neutrophil-to-high-density lipoprotein-C ratio (NHR) is a combined indicator of both inflammation and lipid metabolism (36). All patients underwent a comprehensive blood analysis at the Mazovian Brodno Hospital Laboratory Diagnostics Department. This analysis provided morphological and biochemical evaluations, including absolute neutrophil and lymphocyte counts, platelet counts, and lipid profiles. All three parameters (NLR, PLR, and NHR) were calculated according to the aforementioned patterns based on the blood sample obtained from a single sample.

Cerebral blood flow was examined after administering 740 MBq technetium-99m hexamethylpropyleneamine oxime ([99mTc]Tc-HMPAO) in a quiet room. The data were acquired with a single-photon emission computed tomography/computed tomography (SPECT/CT) scan (Symbia T6, Siemens) on a dual-head gamma camera with a low-energy, high-resolution parallel-hole collimator. The step-and-shoot acquisition mode was used. Sequences of 128 frames on a 128 × 128 matrix were used (64 projections per head, 30 s per projection). The photopeak was set at 140 keV with a 10% window on each side. Repetitive reconstruction (eight iterations, eight subsets, 7 mm Gauss filter), scatter correction, and computed tomography (CT) attenuation correction were performed. The post-processing was examined using Scenium software (Siemens Medical Solutions USA, Inc., Malvern, PA, USA). The SPECT ROIs were pre-planned using Scenium software (an integral part of the Siemens workstation) based on the T1-weighted MRI images of a standard brain dataset. The analysis and definition of subregions were based on a program offered by Siemens Healthineers (SCENIUM, Syngovia).

Software aided in assessing the human brain scans, enabling automated analysis by quantifying mean pixel values within the standard ROI. This also allowed for comparison with existing databases using the healthy control group and the reference parameters derived from these databases, which were derived from SPECT studies, e.g., the calculation of uptake ratios between ROIs and subtraction between two functional scans. The database contained the defined regions and subregions of the brain and the related radiopharmaceutical accumulation values. The data bank was created from images for which reconstructions of Flash3D and CT-based attenuation revision were executed, and intensity normalization was based on the brainstem and the whole brain, respectively. All databases comprised 20 HMPAO-SPECT scans of asymptomatic control individuals aged 64–86 years from a mixed population of men and women. The ROIs used in the Database Comparison (37) were defined on a high-resolution T1 MRI volume scan. In the Database Comparison edition, the statistics are displayed and computed on voxel-by-voxel grounds.

The Database Comparison computed the standard number deviations from each voxel mean value, where the standard deviation and mean values were obtained from the corresponding voxel in the control group brain scans. According to this model, these statistics follow a T-distribution.

The differences in radiopharmaceutical accumulation in the selected ROIs were compared to the database, and these values were reflected in SD. Statistically significant differences in the radiopharmaceutical distribution in the selected ROI are considered if the accumulation exceeded 2 SD values. This method was used in the SCENIUM program (Siemens). The analysis was based on the SD value assessment. The total minimum and maximum counts were automatically measured in each ROI of the investigated brain SPECT examination and were compared using Scenium with measurements from the standard brain SPECT datasets. All comparisons were automatically presented as SDs. This parameter, taken from the ROIs, was evaluated statistically in multiple brain locations. The sizes and shapes of the SPECT-examined brain scans were calibrated per the same parameters as the standard brain scan received from the dataset. The pre-planned ROIs were extrapolated to the SPECT images of the assessed brains.

Finally, the total minimum and maximum counts were automatically assessed in each ROI of the investigated brain during the SPECT examination. Subsequently, they were differentiated with Scenium using measurements from standard brain SPECT datasets. All data were evaluated by an experienced nuclear medicine specialist.

This study assessed many ROIs; however, reduced activity emerged in the thalamus and insula. It has been proposed that decreasing thalamic activation through ascending projections from the brainstem may lead to postural instability in PSP (38). Patients who showed negative results on the [11C]Pittsburgh Compound-B (PIB)-PET (PIB-PET) scans exhibited two primary groups of decreased thalamus metabolism, extending toward the mesencephalon and diencephalon (39). In the insula, imaging revealed gray matter loss in the premotor cortices, supplementary motor area, and insula in the CBS pathologic groups (40). Decreased [11C]UCB-J binding has been observed in the insula, among other areas, in both PSP and CBD patients (41).

### Statistical analysis

The collected data were analyzed using Statistica software (version 13.1, Dell Inc., Statsoft). Data distribution was assessed using the Shapiro-Wilk test. Due to normal distribution, all parameters are expressed as means with standard deviations (SD) and 95% confidence intervals (95% CI). For group comparisons, we used the Student's t-test. Further analysis of the possible correlations in each group of patients between biochemical parameters (NLR, PLR, and NHR) and perfusion in the thalamus and insula was performed using Pearson's correlation coefficient. In the final determination of statistical significance, a p-value of 0.05 was used.

## Results

### Biochemical parameters

The mean age of CBS and PSP patients was similar (72.8 vs. 72.9 years), but the mean age of patients with PD was younger (64.0 years), with a p-value of 0.0575.

The NLR, as a marker of systemic inflammation, also showed similar mean values across all groups: CBS (2.5), PSP (2.3), and PD patients (2.4). Group comparison implies that, based on NLR alone, there was no strong evidence to suggest a difference in systemic inflammation between the groups (p = 0.8635).

The second marker of inflammation, PLR, also exhibited mean values within a comparable range (CBS: 164.5 vs. PSP: 145.4 vs. PD: 124.9; p = 0.2584) in analyzed groups of patients.

The third inflammation marker, NHR, which can also potentially relate to oxygenation status, showed a non-significant p-value (0.7335) compared to its values for CBS, PSP, and PD patients (Table 1).

**Table 1:** Descriptive statistics and subgroup comparison.

**Parameters**	**CBS (N** = **19)**		**PSP (N** = **37)**		**PD (N** = **15)**		**p**
	**Mean**	**SD**	**Mean**	**SD**	**Mean**	**SD**	
Age	72.8	7.2	72.9	6.3	64.0	10.8	0.0575
**Biochemical parameters**							
NLR	2.5	1.6	2.3	0.8	2.4	0.9	0.8635
PLR	164.5	77.9	145.4	54.9	124.9	49.1	0.2584
NHR	0.08	0.05	0.08	0.04	0.09	0.04	0.7335
**SPECT**							
Thalamus R	−4.4	2.4	−4.4	2.2	−1.7	1.7	0.0003
Thalamus L	−4.2	1.8	−4.1	1.9	−1.9	1.5	0.0008
Insula R	−1.9	1.8	−1.5	2.0	0.2	1.8	0.0049
Insula L	−3.2	2.6	−3.1	3.2	−0.7	1.9	0.0167

SD, standard deviation; p, p-value for Kruskal–Wallis test.

### SPECT

There were statistically significant differences between the tauopathic atypical parkinsonisms and PD; simultaneously, crucial differences between PSP and CBS were not found in terms of SPECT values in the compared regions (right thalamus p = 0.003, left thalamus p = 0.008, right insula p = 0.0049, left insula p = 0.0167) as shown in Table 1. After post-hoc analysis, we observed no statistically significant differences between CBS and PSP patients in terms of SPECT measurements for any of these regions. However, these values differed significantly for PD patients relative to CBS and PSP patients (Table 2).

**Table 2:** PD patients in relation to CBS and PSP patients.

	**CBS vs. PD**	**PSP vs. PD**
Thalamus R	0.0024	0.003
Thalamus L	0.0036	0.0012
Insula R	0.0064	0.0160
Insula L	0.0253	0.0349

#### NHR and the insula

**Figure 1:**
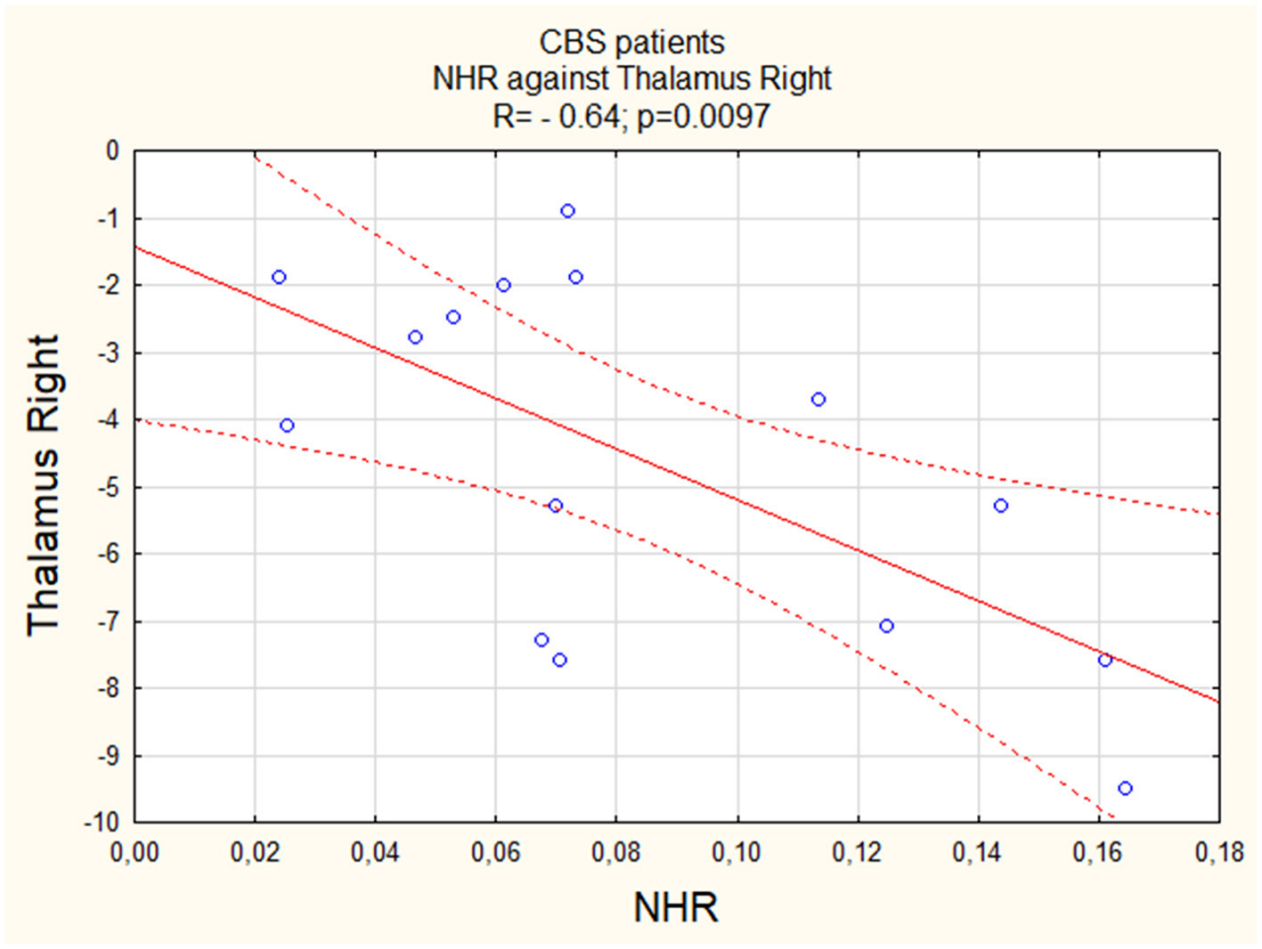
The correlation between perfusion of the left insula and NHR in CBS.

**Figure 2:**
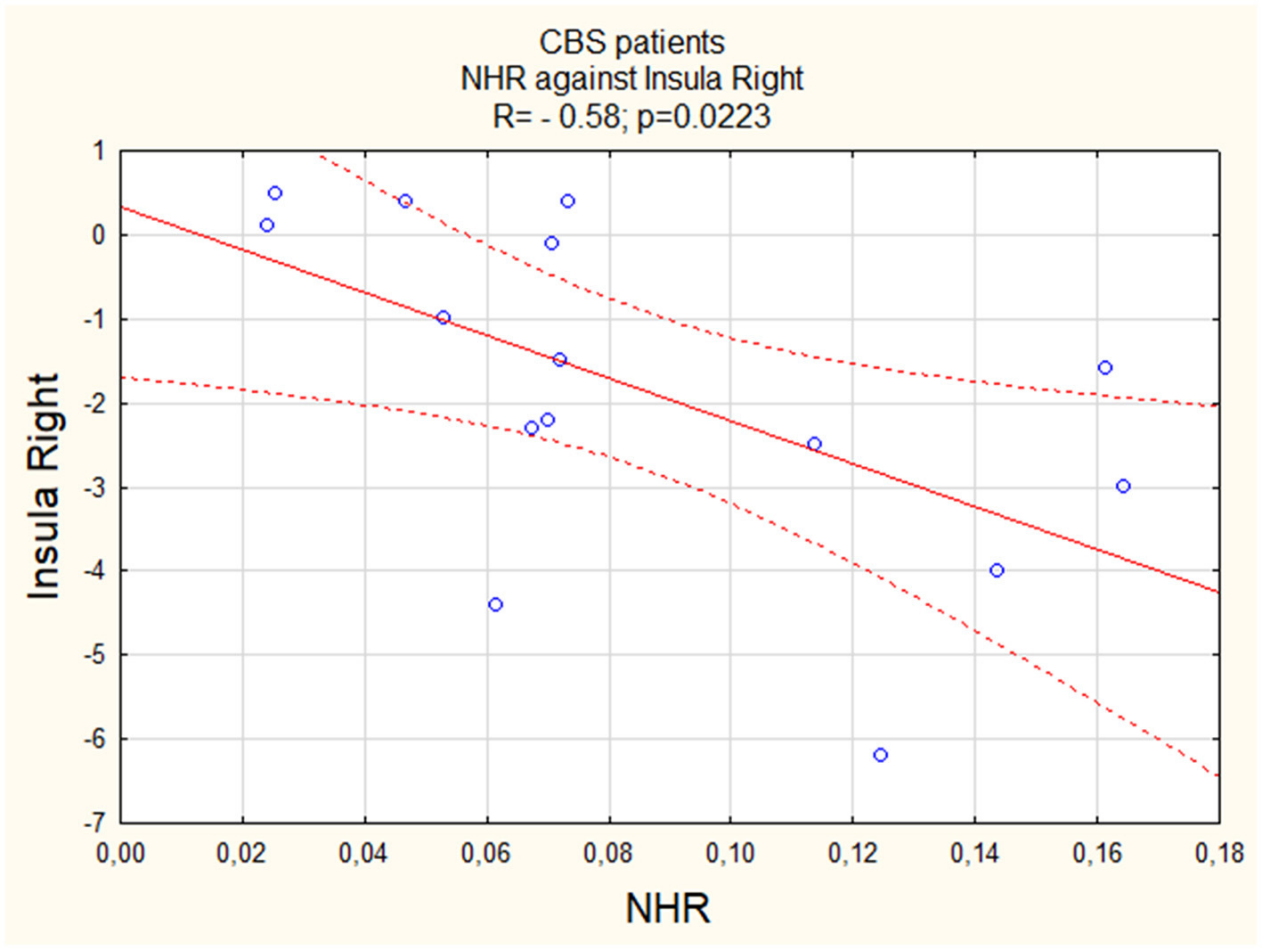
The correlation between perfusion of the right insula and NHR in CBS.

We observed a negative linear correlation between NHR and activity of both the left (Insula L; R = −0.59, Figure 1) and right (Insula R; R = −0.58, Figure 2) insula regions, suggesting that higher values were associated with lower activity levels in these areas. For both PSP patients and PD patients, correlations were insignificant (p > 0.05).

#### NHR and the thalamus

**Figure 3:**
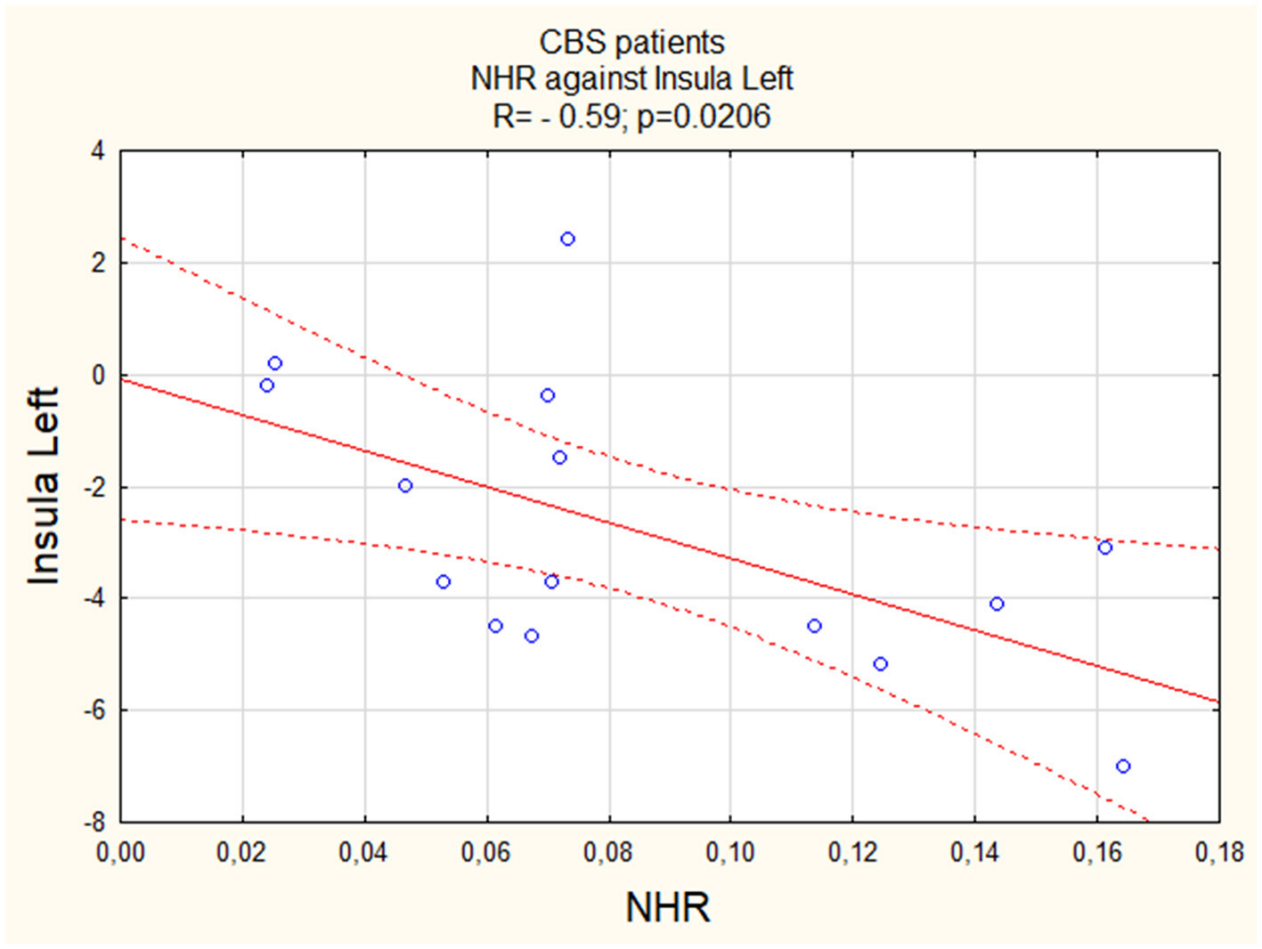
The correlation between perfusion of the right thalamus and NHR in CBS.

**Figure 4:**
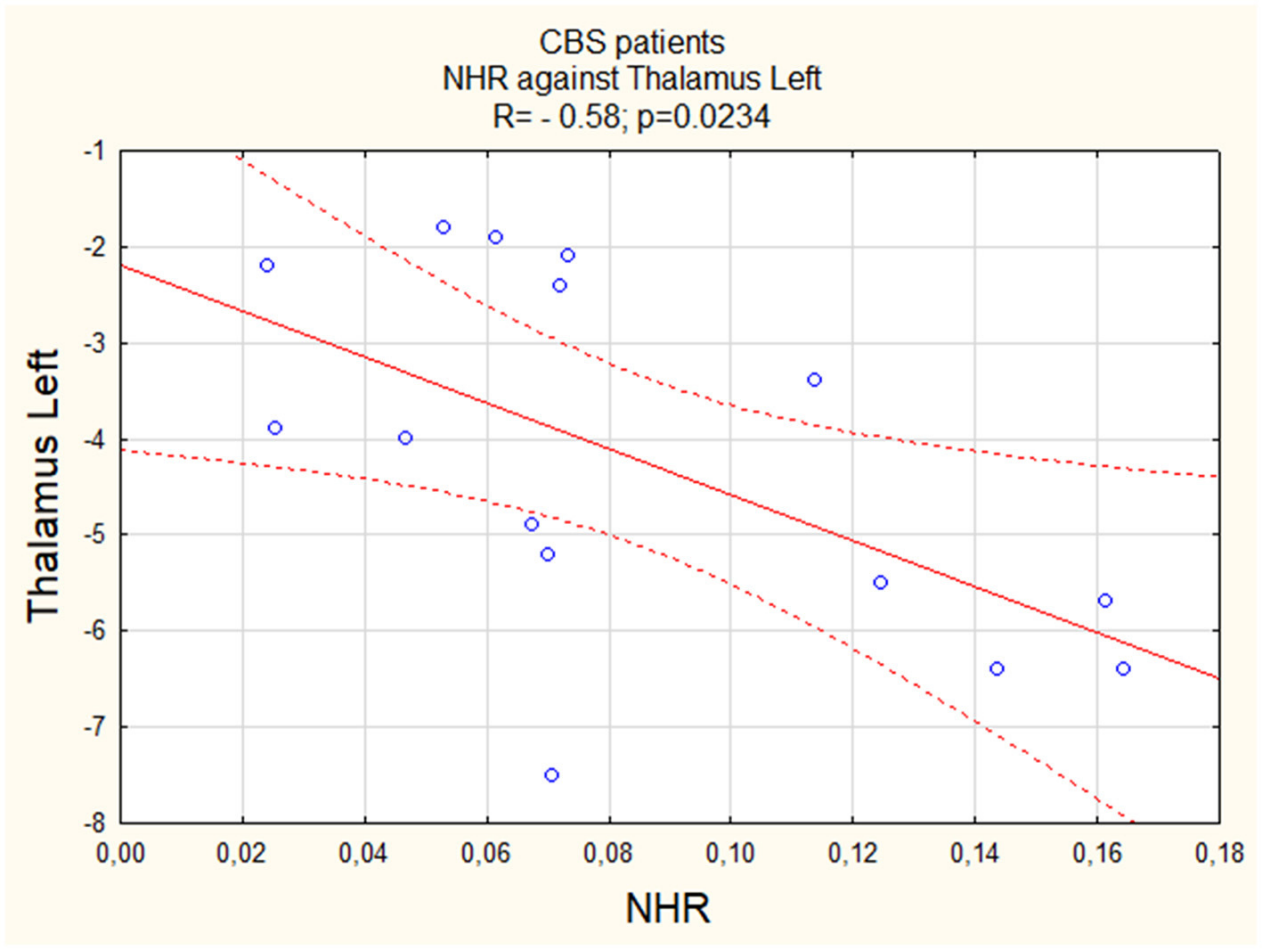
The correlation between perfusion of the left thalamus and NHR in CBS.

Similar to the insula, we observed a negative linear correlation between NHR and activity in both the left (Thalamus L) and right (Thalamus R) thalamus regions, with a relatively stronger correlation in the right thalamus (R = −0.64, Figure 3 vs. R = −0.58, Figure 4). For both PSP patients and PD patients, correlations were insignificant (p > 0.05).

## Discussion

A statistically significant negative correlation was observed between NHR and perfusion in the insula and thalamus in patients with CBS (Table 3). The correlation was not detected among patients with PSP and PD. The group of individuals suffering from PD was, on average, ~9 years younger than the other two groups. This was due to the fact that as Parkinson's disease progresses, the average lipid levels decrease, which could potentially disrupt the comparison of NHR values between the groups. (42) NHR is a peripheral inflammatory factor likely associated with inflammation caused by extensive microglial activation, which is linked to this phenomenon. The inflammation and microglial activation appear to be key factors in neurodegeneration in atypical parkinsonism and other neurodegenerative diseases. The presence of a highly oxidizing, pro-inflammatory environment combined with a high concentration of microglia makes dopaminergic neurons in the substantia nigra especially susceptible to neuroinflammation (43). Glial cells are crucial for cholesterol synthesis and metabolic balance in the brain, where a particle resembling HDL containing apolipoprotein E (ApoE) facilitates cholesterol transport. Astrocytes and microglia are responsible for the secretion of ApoEs found in these HDL-like particles (44). There is a strong possibility that dyslipidemia is causally related to impaired blood–brain barrier (BBB) function (45). Higher HDL-C levels are linked to reduced BBB permeability (44). Breakdowns in both the structure and performance of the BBB occur naturally with aging. However, this deterioration is significantly amplified in numerous neurodegenerative conditions and serves as a prominent sign of cognitive impairment or even dementia (46).

**Table 3:** Correlation between NHR and insula and thalamus perfusion for CBS, PSP, and PD patients.

	**CBS**		**PSP**		**PD**	
	**Rp**	**p**	**Rp**	**p**	**Rs**	**p**
NHR and insula L	−0.59	0.0206	0.14	>0.05	0.13	>0.05
NHR and insula R	−0.58	0.0223	0.15	>0.05	−0.39	>0.05
NHR and thalamus L	−0.58	0.0234	0.14	>0.05	−0.49	>0.05
NHR and thalamus R	−0.64	0.009	0.11	>0.05	−0.31	>0.05

Rp, Pearson coefficient; Rs, Spearman coefficient; p, p-value for Pearson or Spearman coefficient correlation.

HDL facilitates reverse cholesterol transport (RCT), a mechanism that extracts excess cholesterol from peripheral tissues and delivers it to liver cells. Therefore, cholesterol is metabolized and eliminated (47). Additional beneficial roles of HDL include anti-inflammatory, antioxidant, and vasodilatory activities. HDL also plays a role in regulating insulin secretion and insulin sensitivity. HDL functions relate to the structure and composition of its particles, which is evident in the varying biological activities of its two primary subclasses, HDL2 and HDL3. Smaller and denser HDL3 particles play a more significant role in cholesterol efflux, whereas larger HDL2 particles are more actively involved in antithrombotic activities (48). The described HDL properties negatively correlate with the proposed mechanisms leading to diseases such as PSP or CBS. Considering the functions of the HDL subclasses, HDL2 appears to be a greater protective factor than HDL3. This only confirms the importance of further research on the relationship between HDL and these tauopathies.

As it turns out, HDL influences the risk of diseases such as AD or FTD, which may clinically manifest. Research has indicated a relationship between higher plasma levels of HDL-C and ApoE levels and a lower risk of dementia (49). Similar to ApoE, Apolipoprotein J (ApoJ) attaches to HDL and HDL-like particles and plays a vital role in cholesterol metabolism in the brain. ApoJ prevents the aggregation of both amorphous and amyloidogenic proteins triggered by stress in various ways. It binds to the hydrophobic sections of aggregated or misfolded proteins, either breaking them down or reducing their toxicity. Additionally, under normal physiological conditions, ApoJ plays a neuroprotective role by blocking the aggregation of Aβ. (50). ApoJ was found to be increased in AD patients (51). HDL defends against cognitive deterioration in AD (52). The behavioral variant FTD (bvFTD) cohort (N = 31) showed reduced levels of HDL compared with the control group (53). The difference in the correlation between CBS and PSP may be attributed to the fact that CBS can be a symptom of many diseases, such as AD or FTD, whereas PSP is a more suggestive clinical diagnosis than CBS (54). There is a significant overlap between the diagnosis of CBS and PSP (55). In FTD, cognitive phenotypes frequently overlap with motor phenotypes, including motor neuron diseases, parkinsonian symptoms, and syndromes such as CBS or PSP (56). Extrapyramidal symptoms combined with apraxia suggest the presence of CBS, which is predominantly associated with Tau disease. In contrast, dementia in the context of FTD with motor neuron disease (FTD-MND) syndrome is mainly linked to TDP-43 pathology (57). AD also might resemble CBS and PSP, but AD is more connected with the Tau protein phosphorylated at threonine 181 (p-tau181) (58). Both markers are potentially present in the CSF.

SPECT examination is widely used in the differential diagnosis of 4R tauopathies (4RT). Research employing the same radiotracer identified perfusion abnormalities in the prefrontal cortex in PSP, but in CBS, the irregularities occurred in the inferior prefrontal, sensorimotor, and posterior parietal cortices. Furthermore, another study demonstrated a more pronounced asymmetry in blood flow in CBS (59). SPECT allows for the distinguishing of CBS patients from PD patients. CBS patients exhibited reduced perfusion in the temporoinsular area, insula, or thalamus (60). The anterior insula's increased functionality was correlated with Interleukin-6 elevation in the serum of the CBS patients (61). The correlation between NHR and perfusion of the insula suggests a potential relationship between systemic inflammation (as reflected by NHR) and reduced activity in the insula, which could contribute to the symptomatology observed in CBS patients, such as motor dysfunction and cognitive impairment. Reduced activity in this region can affect, among other functions, sensory perception, motor control, and regulation of consciousness. It is worth adding that impaired judgment, lack of empathy, and impulsivity/disinhibition are clinical characteristics of the behavioral variant of bvFTD. These deficits are consistent with the roles of the anterior insula region (62). Patients with AD express atrophy of the insular cortex, which may reflect typical symptoms such as progressive memory loss, diminished activities of daily life, language impairment, motor skill disorders, and loss of perception (63, 64). Additionally, the thalamus, among other structures, is likely responsible for sleep disturbances observed in individuals with AD (65). Individuals with bvFTD and AD exhibit marked bilateral volume losses in the thalamus (66, 67). Differential thalamic involvement, identified through diffusion measurements, may be useful in distinguishing PSP from CBD. In PSP, the anterior and medial thalamic nuclei were found to be more affected, whereas, in CBD, the motor thalamus region was predominantly affected (60). To the best of our knowledge, the correlation between the perfusion of these mentioned ROI and NHR levels has not been explored previously.

It should be noted that NLR and NHR are widely used as peripheral inflammatory indicators in other diseases than 4RT. The average NLR value in the PSP group was notably higher than that in both the PD group and healthy control subjects (68). As a reference point for the study group, we adopted laboratory norms for neutrophils, blood platelets, and HDL. Moreover, the NLR can predict mortality in the general population and is significantly associated with higher overall mortality rates (69). Studies have claimed that inflammation plays a pivotal role in PD pathogenesis, assessing, among other things, NLR and NHR contributions. In the PD group, there was a significant increase in neutrophil count, NHR, and NLR. In contrast, hypertension, body mass index, and lymphocyte count, as well as total cholesterol levels, triglycerides, LDL cholesterol, and uric acid were substantially reduced compared to the control group. Meanwhile, correlation analysis revealed that the NHR was significantly negatively associated with disease duration. The NHR has significant predictive power for PD and is intricately linked to the disease's duration. These findings suggest that the NHR could be a superior indicator of long-term clinical outcomes in PD patients compared with the NLR (70, 71). There are many reports about neutrophils and HDL having a significant association with cardiovascular diseases (72, 73).

Additionally, during acute cardiovascular events, abundant neutrophil aggregates lead to increased expression of local inflammatory mediators, which heightens inflammation and exacerbates the condition. HDL-C supports endothelial function and blood viscosity and possesses anti-atherosclerotic properties (72). The NHR can be used, for example, to evaluate survival prognosis in ischemic strokes. The NHR is assumed to facilitate the identification of suitable symptomatic therapies for patients (73).

However, there are no data on the influence of the NHR on the development of symptoms, selection of more appropriate therapy, and, consequently, survival rate in the context of 4RT tauopathies. PSP and CBS contributed to the elevation of the NLR. A significant difference in the NLR increase was observed exclusively in PSP, whereas the rise in NLR within CBS cases was less marked and lacked significant differences (27). It seems that, as seen in the case of PD and vascular diseases, a proper understanding of the precise mechanism of action of peripheral inflammatory markers such as NLR, PLR, and NHR will not only allow better prediction of survival duration but also enable the selection of the appropriate therapies. Consequently, this approach could extend the survival time of patients suffering from 4RT.

Neuroinflammatory PSP pathogenesis was also associated with a significant increase in pro-inflammatory and microglia-related cytokines (IL-1β, IL-6, and TNF-α) and IL-4 (74, 75). Additionally, PSP individuals expressed elevated IL-2 levels associated with malfunctioning peripheral inflammation (74). One study postulated that patients with PSP express higher levels of TGFβ in cortical areas, as well as IL-1β, which is more concentrated in the substantia nigra (76). There was a correlation found between IL-6 serum levels and PSP severity (77). Data related to the CBS interleukin profile were not found. However, CBS can be a symptom of FTD. In individuals with autosomal dominant FTD, elevated IL-6 levels were linked to a more rapid functional decline, while TNFα was associated with both this deterioration and temporal lobe atrophy (78).

This study primarily evaluated the utility of assessing peripheral inflammation in the context of diagnosing CBS or PSP. It is especially crucial because these diseases exhibit considerable phenotypic overlap; therefore, an additional diagnostic tool will be extremely important for establishing an accurate diagnosis (55). Identifying a specific target within the pathomechanism of CBS may provide the opportunity to find effective targeted future therapies for patients suffering from the syndrome. Both inflammation and brain hypoperfusion caused by modifiable factors, e.g., atherosclerosis, narrowing of the blood vessels, chronic infections, and many environmental factors, can be mitigated through appropriately chosen pharmacotherapy, which can impact the quality of life of these subjects in the future.

## Limitations

The research was constrained by several limitations. The study lacked neuropathological evaluation because all patients are currently alive. Additionally, the cohort is relatively small, comprising 71 subjects, of whom 19 were diagnosed with CBS, 37 with PSP, and 15 with PD. The relatively small proportion of patients and the CBS cohort was insufficiently balanced in terms of gender. These two aspects were associated with the fact that both CBS and PSP are rare diseases; moreover, patients affected by the clinical entities suffer due to limited mobility. Additionally, PD patients' average age was lower than in the case of the other study groups. In this study, particular CBS and PSP and specific subclasses of HDL were not acknowledged. Assessments were based on a singular examination. Due to the rapid clinical deterioration, conducting a reliable subsequent evaluation of these diseases was not possible. The study is based on non-specific, easily accessible diagnostic tools.

## Conclusion

This study showed a negative correlation between the NHR increase and perfusion concerning the thalamus and insula via SPECT examination in the context of CBS. The NHR is a non-specific indicator of peripheral inflammation. The NHR index may be a good indicator of hypoperfusion due to the negative correlation of HDL with atherosclerotic plaques and neutrophils, which significantly increases the risk of blood vessel stenosis and, consequently, hypoperfusion. It appears that a high level of HDL (which is negatively correlated with the NHR) has a protective effect on the BBB and, consequently, on the degeneration process. Decreased perfusion may suggest the evolution of neurodegenerative changes. The same correlation was not observed in the case of the PSP and PD. This finding may arise from the fact that CBS can occur in a more heterogeneous group of pathologies when compared to PSP or PD. Additionally, this may suggest possible differences in the pathomechanism of these two diseases. There is currently a lack of efficient treatment options, and an analysis of the course of inflammation may enable further studies into possible effective treatments. Additional studies involving larger groups of patients with neuropathological verification are necessary.

## Data Availability

The raw data supporting the conclusions of this article will be made available by the authors, without undue reservation.
